# Relationship between ABO blood group and risk of venous thrombosis in cancer patients with peripherally inserted central catheters: A protocol for systematic review and meta-analysis

**DOI:** 10.1097/MD.0000000000023091

**Published:** 2020-11-06

**Authors:** Fangjing Liu, Qiang Zhang, Li Rao, Jifang Song

**Affiliations:** Department of Oncology, Army Medical Center of PLA, Chongqing, China.

**Keywords:** ABO blood group, venous thrombosis, cancer, peripherally inserted central catheters, protocol, systematic review, meta-analysis

## Abstract

**Background:**

Peripherally inserted central catheter (PICC) is closely related to venous thromboembolism (VTE). It is a common complication of hospitalized patients, and its etiology is complex. How to prevent the occurrence of VTE is the focus of clinical work. In order to provide basis for individual prevention and accurate treatment of VTE, the purpose of this study was to explore the relationship between ABO blood group and the risk of VTE in cancer patients associated with PICC through meta-analysis.

**Methods:**

Electronic databases, including Embase, Cochrane Library, Pubmed, Chinese databases SinoMed, Chinese National Knowledge Infrastructure (CNKI), Chinese Scientific Journals Database (VIP), and Wanfang Data, were searched for case-control studies of ABO blood group and PICC-related VTE in cancer patients. The languages were limited to Chinese and English. Two reviewers were responsible for the selection of the study, the extraction of data and the evaluation of the quality of the research. All statistical analyses were carried out with Review Manager 5.3 and Stata 14.0.

**Results:**

The results of this meta-analysis would be published in peer-reviewed journals.

**Conclusion:**

This study provided evidence to support the relationship between ABO blood group and the risk of PICC-related VTE in cancer patients.

**OSF Registration number:**

DOI 10.17605/OSF.IO/6DPFG

## 1 Introduction

Peripherally inserted central venous catheter (PICC) is widely used in chemotherapy and nutritional support for tumor patients.^[1–4]^ The infusion of chemotherapeutic drugs through PICC can reduce the risk of drug extravasation and the stimulation of peripheral blood vessels, avoid the pain caused by repeated puncture, and greatly improve the quality of life of cancer patients.^[5–7]^ However, the blood of tumor patients is mostly in a state of hypercoagulability or vascular endothelial damage due to the influence of long-term chemotherapeutic drugs. The incidence of PICC-related venous thromboembolism (VTE) significantly increased, and became one of the most common factors of PICC catheter wall plug.^[8–10]^

Many studies revealed that patients with non-O blood group have an increased risk of VTE.^[11,12]^ A number of studies proved that ABO blood group has an obvious impact on coagulation function, because ABO blood group is the main determinant of plasma von Willebrand factor (vWF) level.^[13–15]^

Overall, about 70% of the changes in vWF/F VIII plasma levels is determined by heredity, of which about 30% is related to individual ABO blood type. It is worth noting that about 25% of individuals with non-O blood groups has high levels of vWF. Many scholars further explore the possible clinical significance of this biological relationship. For example, whether ABO blood type may be a factor that affects the risk of bleeding or arterial / VTE thrombosis, etc.

Previous studies displayed that people with nontype 0 blood have a remarkable higher risk of VTE and coronary heart disease than those with type O blood.^[16]^ Gándara et al^[17]^ observed that the incidence of VTE is high in people with non-O blood group, and considered that non-O blood is a risk factor for VTE. Isabel et al^[18]^ discovered that high VII level and non-O blood group are independent risk factors for VTE, and should be considered in the evaluation of thrombotic hemophilia.

The common risk factors of PICC-VTE are well known, but there are few studies on the relationship between ABO blood group and them. At present, the relationship between ABO blood group and PICC-related VTE in cancer patients is still controversial.^[19,20]^ In this paper, in order to better clarify the possible relationship between ABO blood group and PICC-related VTE in cancer patients, and to provide a basis for individualized prevention and accurate treatment of ABO blood group and PICC-related VTE in cancer patients, we collected a case-control study on the relationship between ABO blood group and the risk of PICC-related VTE in cancer patients for meta-analysis.

## 2 Methods

### 2.1 Study registration

The protocol was registered in Open Science Framework (registration number: DOI 10.17605/OSF.IO/6DPFG). This systematic review and meta-analysis will be reported inconformity with the preferred reporting items for systematic reviews and meta-analysis protocols (PRISMA-P) 2015.^[21]^

### 2.2 Ethic

The review does not involve the assessment of patients’ individual information or rights, so there is no need to obtain approval from an ethical institution.

### 2.3 Inclusion criteria

Studies would be included in this meta-analysis based on following criteria:

1. Study types: All case-control studies related to ABO blood type and PICC-related VTE in cancer patients susceptibility should be included. The publication of literatures is unrestricted.
2. Participant type: Tumor patients implanted with PICC should be included in the meta-analysis. Not subject to age, sex, or national restrictions.
3. Outcome: PICC-related VTE risk comparisons.

### 2.4 Exclusion criteria

According to the following criteria, studies should be excluded from the meta-analysis: Conference summaries, incomplete data studies, repeated published studies, and case series.

### 2.5 Search strategy

Embase, Cochrane Library, Pubmed, Chinese databases SinoMed, Chinese National Knowledge Infrastructure (CNKI), Chinese Scientific Journals Database (VIP), and Wanfang Data were searched. The details of PubMed's search strategy are shown in Table 1, including all search terms, while similar search strategies are applied to other electronic databases.

**Table 1:** Search strategy in PubMed database.

Number	Search terms
#1	Catheterization, Central Venous[MeSH]
#2	Central Venous Catheterization[Title/Abstract]
#3	Venous Catheterization, Central[Title/Abstract]
#4	Catheterization, Central[Title/Abstract]
#5	Central Catheterization[Title/Abstract]
#6	Catheterizations, Central[Title/Abstract]
#7	Catheterizations, Central Venous[Title/Abstract]
#8	Central Catheterizations[Title/Abstract]
#9	Central Venous Catheterizations[Title/Abstract]
#10	Venous Catheterizations, Central[Title/Abstract]
#11	Peripherally inserted central catheter[Title/Abstract]
#12	PICC[Title/Abstract]
#13	Central Venous Catheters[MeSH]
#14	Central Venous Catheter[Title/Abstract]
#15	Catheter, Central Venous[Title/Abstract]
#16	Catheters, Central Venous[Title/Abstract]
#17	Venous Catheter, Central[Title/Abstract]
#18	Venous Catheters, Central[Title/Abstract]
#19	or/1-18
#20	Venous Thromboembolism[MeSH]
#21	Thromboembolism, Venous[Title/Abstract]
#22	VTE[Title/Abstract]
#23	or/20-22
#24	ABO Blood-Group System[MeSH]
#25	ABH Blood Group[Title/Abstract]
#26	ABO Factors[Title/Abstract]
#27	Blood Group H Type 1 Antigen[Title/Abstract]
#28	H Blood Group[Title/Abstract]
#29	H Blood Group System[Title/Abstract]
#30	ABH Blood Groups[Title/Abstract]
#31	ABO Blood Group System[Title/Abstract]
#32	ABO Blood-Group Systems[Title/Abstract]
#33	ABO Factor[Title/Abstract]
#34	Blood Group, ABH[Title/Abstract]
#35	Blood Group, H[Title/Abstract]
#36	Blood Groups, ABH[Title/Abstract]
#37	Blood Groups, H[Title/Abstract]
#38	Blood-Group System, ABO[Title/Abstract]
#39	Blood-Group Systems, ABO[Title/Abstract]
#40	Factor, ABO[Title/Abstract]
#41	Factors, ABO[Title/Abstract]
#42	H Blood Groups[Title/Abstract]
#43	System, ABO Blood-Group[Title/Abstract]
#44	Systems, ABO Blood-Group[Title/Abstract]
#45	or/24–44
#46	19 and 23 and 45

### 2.6 Data collection and analysis

#### 2.6.1 Selection of studies

**Figure 1:**
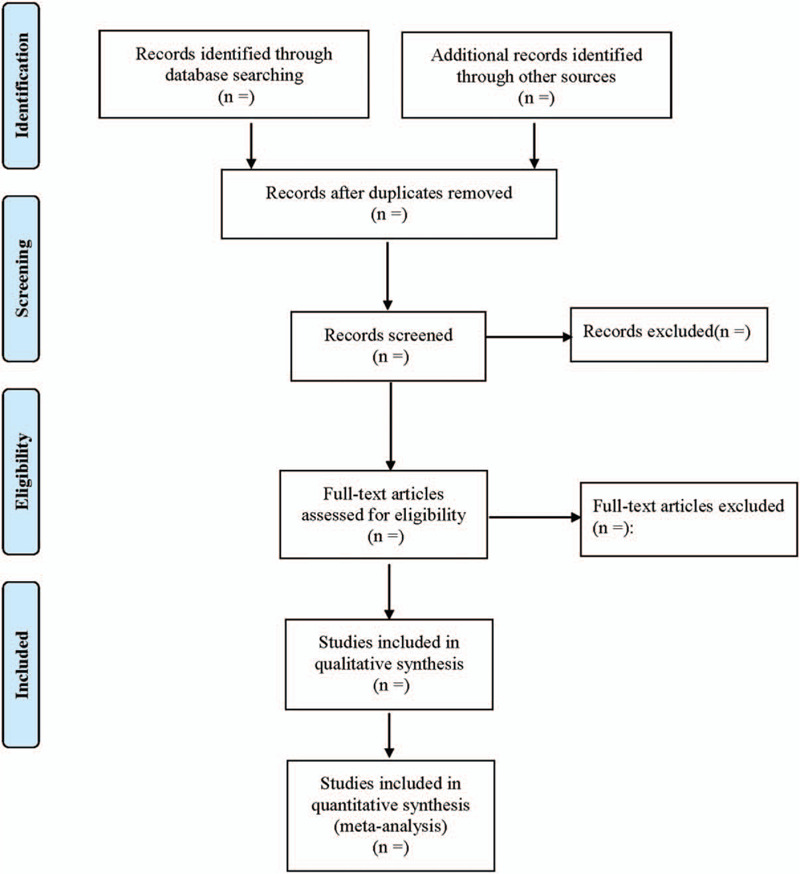
Flow diagram of study selection process.

Two researchers read the questions and abstracts alone to screen relevant literatures. By reading the full text, the research design, inclusion and exclusion criteria, comparison and judgment of the research results of the relevant literature were evaluated. According to the inclusion and exclusion criteria, choosing whether to include the file in the meta-analysis. If there is a dispute, the third researcher would re-evaluate and decide whether to include the analysis. The screening flow chart of this study is displayed in Figure 1.

#### 2.6.2 Data extraction

We extracted data from literatures that conform to the meta-analysis. The data includes the first author, year of publication, country, race of each study population, number of cases and controls, sex, average age, ABO blood type, and number of cases of thrombosis, etc.

#### 2.6.3 Study quality assessment

Two researchers independently applied Newcastle-Ottawa scale to evaluate literatures included in the analysis, and adopted third-party opinions if there exist any differences.^[22]^ According to the evaluation content of Newcastle-Ottawa scale scale, they were divided into 8 items and 9 points, and each score was 1 point.^[23]^

(1) The choice of the study population. Is the case selection appropriate? Is it representative? Is the choice of the control group the same population as the case group? How to determine the control group? One point per point, and a total of 4 points.
(2) Comparability among groups. Does the study design and statistical analyses are taken into account the comparability between the case group and the control group, and whether the confounding factors are controlled? Age, race, region, gender, and sample structure were all regarded as important confounding factors, and those who controlled 1 item scored 1 point, with a total of 2 points.
(3) The determination of exposure factors. How to determine the exposure factors? Is the same method used to determine the exposure factors in the case group and the control group? What is the nonresponse rate of the two groups? One point per point, and a total of 3 points.

Literatures with a total score of more than 6 are considered to be high quality.

#### 2.6.4 Dealing with missing data

The research on the defects of the original data. We contacted the author by email and asked for the original data. If the original data is not available, then we would analyze the existing data.

### 2.7 Statistical analysis

RevMan 5.3 (provided by Cochrane Collaboration) and Stata 14.0 (STATA Corp, College Station, TX, USA) were applied for statistical analysis. Odds ratio is the statistic of effect analysis, and each effect dose provides its 95% confidence interval. The heterogeneity among the included results was analyzed by χ^2^ test (the test level was α = 0.1). Meanwhile, combining with *I*^2^ to quantitatively judge the size of heterogeneity. If there was no statistical heterogeneity among the results, the fixed effect model was adopted for meta-analysis. If there is statistical heterogeneity among the results of each study, the source of heterogeneity is further analyzed. After excluding the influence of obvious clinical heterogeneity, random effect model is adopted for meta-analysis. The level of meta-analysis was set as α = 0.05, and the obvious clinical heterogeneity was treated by subgroup analysis or sensitivity analysis, or only descriptive analysis.

### 2.8 Subgroup analysis

According to patient race, sample size, tumor type and so on, we carried out subgroup analysis.

### 2.9 Sensitivity analysis

Through the study of large weight of elimination effect, the sensitivity analysis was conducted to test the stability of the results of meta-analysis.

### 2.10 Assessment of publication biases

If more than 10 studies are included, a funnel chart would be applied to assess the report bias.^[24,25]^ In addition, publication bias was further quantitatively evaluated by Egger and Begg test.

### 2.11 Grading the evidence quality

We utilized Grading of Recommendation Assessment, Development and Evaluation method to evaluate the evidence quality of the obtained results.^[26]^

## 3 Discussion

PICC provides an effective venous channel for patients undergoing chemotherapy and relieves the pain caused by repeated venipuncture.^[27]^ However, many PICC complications can be observed in tumor patients, of which thrombus is an important consequence.^[28,29]^ Tumor is a high risk factor for thrombosis, and the risk of thrombosis in tumor patients is 4 to 7.5 times higher than that in the general population.^[9,10]^

ABO blood group system is closely related to the level of vWF^[30,31]^ that is a polymer with different molecular weight secreted by endothelial cells and megakaryocytes. As a protective carrier of coagulation factor VIII, it can mediate the adhesion between platelets and subendothelial collagen, and the aggregation between platelets and platelets, thus promoting thrombosis. VWF would be cleaved under the catalysis of metalloproteinase ADAMTS13 and eventually become a nonfunctional proteolytic fragment. The mechanism of ABO antigen system that affects thrombosis may be that its antigen exists on vWF through oligosaccharide chain. Different oligosaccharide chains of different blood groups affect the steric hindrance and charge effect of vWF structure, the effect of metalloproteinase ADAMTS13, and the rate of cleavage.^[32,33]^ Furthermore, vWF initially existed in the form of a large complex, and when the endothelium was damaged, it folded tightly. The stacked vWF would become loose under the action of blood flow, exposing the acting groups and producing biological activity. The difference of oligosaccharide chain may affect the change rate of vWF structure.

Early prevention and intervention strategies are very important to reduce the incidence of PICC-related VTE for cancer patients. Determining the genetic composition of PICC-related VTE in cancer patients is a key area of VTE research. The identification of VET-related genes affects the understanding of its molecular and mechanism level, treatment and prevention. Therefore, in order to identify high-risk patients and design targeted treatment strategies to prevent serious complications in the future, we explored the ABO blood group. At present, although there are studies on the relationship between ABO blood group and PICC-related VTE in cancer patients risk. There is no systematic assessment of the cumulative evidence of this connection. We will conduct a systematic review and meta-analysis to clarify the relationship between ABO blood group and PICC-related VTE in the susceptibility of cancer patients.

## Author contributions

**Data collection:** Qiang Zhang

**Funding support:** Qiang Zhang

**Literature retrieval:** Fangjing Liu

**Software operating:** Fangjing Liu

**Supervision:** Li Rao and Jifang Song

**Writing – original draft:** Qiang Zhang, Fangjing Liu and Li Rao

**Writing – review & editing:** Li Rao and Jifang Song
